# Challenges in adjustability and mechanism: analysis on results obtained with maxillary appliances for moderate obstructive sleep apnea

**DOI:** 10.1186/s13063-025-08944-1

**Published:** 2025-07-03

**Authors:** Thibault Canceill, Laura Pascalin, Anne Decotte, Guillaume De Bonnecaze

**Affiliations:** 1 Odontology Department, Toulouse Health Faculty, Paul Sabatier University, 3 Chemin Des Maraichers, Toulouse, 31400 France; 2 Odontology Department, Toulouse University Hospital, 2 Rue de Viguerie, Toulouse, 31000 France; 3 InCOMM (Intestine ClinicOmics Microbiota & Metabolism), UMR1297 Inserm, Université Toulouse III, French Institute of Metabolic and Cardiovascular Diseases (i2MC), 1 Avenue Jean Poulhès, Toulouse, 31400 France; 4 Otorhinolaryngology Department, Toulouse University Hospital, 24 Chemin de Pouvourville, Toulouse, 31400 France

**Keywords:** Sleep apnea, Oral appliance, Mandibular advancement device

## Abstract

Comment on “Comparative evaluation of the efficacy of customized maxillary oral appliance with mandibular advancement appliance as a treatment modality for moderate obstructive sleep apnea patients.”

The recent article by Belkhode et al. presents the results of a randomized controlled trial comparing two oral appliances—a customized maxillary oral appliance (CMOA) and a mandibular advancement device (MAD)—in the treatment of moderate obstructive sleep apnea (OSA) [1]. This study is timely and relevant as it addresses a critical need for alternatives to continuous positive airway pressure (CPAP), a gold-standard treatment [2] that, despite its efficacy, faces compliance issues among patients due to discomfort and inconvenience [3].

Belkhode et al. have proposed a robust methodology, with assessments of apnea/hypopnea index (AHI), oxygen saturation, and various sleep quality parameters over a period of 3 months. The inclusion of objective polysomnography measurements is especially commendable, as it ensures high accuracy and reliability in capturing treatment effects, which is essential for drawing clinically meaningful conclusions in sleep medicine [4].

Custom-made dual-block mandibular advancement devices have gained popularity as a non-invasive treatment option for patients with mild to moderate OSA. They are recommended by the European Respiratory Society as a valuable alternative for CPAP in low to moderate obstructive sleep apnea [5]. However, tolerance to MAD treatment can be impacted by various side effects, including temporomandibular joint (TMJ) discomfort, tooth pain, salivation disorders, and gum irritation [6]. Further literature indicates that, while MADs offer a viable alternative to CPAP, the predictability of the treatment and its efficacy can vary significantly [7] depending on the patient’s anatomical and physiological response, as well as individual tolerance to the device. Understanding the variability in patient tolerance is crucial, as it influences both compliance rates and overall effectiveness in managing OSA symptoms. In light of these tolerance issues, the introduction of a customized maxillary oral appliance (CMOA) in Belkhode et al.’s study appeared to be interesting, as it may provide an alternative with potentially fewer side effects, thereby improving patient tolerance and comfort. On this point of tolerance, which is critical for evaluating long-term adherence to treatment, the study lacks data. Incorporating evaluations under drug-induced sleep endoscopy could have provided insights into the device’s impact on airway patency, particularly regarding lingual protraction and gag reflex due to the appliance’s extension over the soft palate.

Moreover, the mechanism by which the new maxillary appliance could significantly improve OSA symptoms is intriguing and raises questions. If merely increasing the vertical dimension with a maxillary appliance was sufficient to alleviate apnea symptoms, it would stand to reason that patients using maxillary splints for bruxism would also experience reduced OSA severity. Yet, such an effect has not been observed in clinical practice, where bruxism splints are not known to impact OSA outcomes. This discrepancy suggests that other factors, possibly related to anatomical changes in airway support or specific design elements for air supply with the CMOA, might be at play.

Another critical consideration is the question of device adjustability. Unlike a MAD, which can be precisely adjusted in terms of mandibular advancement to optimize efficacy and comfort, it is unclear how adjustments could be made to the CMOA once it has been fabricated. MADs allow incremental tuning to suit individual patient needs over time, which is essential for both comfort and effectiveness in managing OSA. With the CMOA, however, if adjustments are required post-fabrication, it appears the only option would be to remake the appliance entirely. This lack of adjustability may limit the practical application of CMOA in a real-world clinical setting, as fine-tuning is often essential in OSA treatment to accommodate each patient’s unique anatomy and therapeutic response. Clarification on this point from the authors would be valuable, as would further exploration of design innovations that could introduce adjustability to CMOA devices.

A point of concern arises in the data presented. On the one hand, there appears to be a discrepancy between the values listed in Tables 1 and 5, specifically with a standard deviation value of 0.068 in Table 1 contrasted against 3.67 in Table 5 for what appears to be the same element. This inconsistency requires clarification as it may affect the interpretation of the study’s outcomes and reliability. On the other hand, while the trial is presented as a randomized controlled study, the emphasis in both the abstract and the main text is primarily on within-group improvements. This analytical focus may distract from the more scientifically robust between-group comparisons, which are the cornerstone of randomized trial designs. The statement suggesting than the CMOA is as efficient than MAD in treating moderate OSA implies therapeutic equivalence. This interpretation is not appropriate given the design and statistical analysis of the study.

In conclusion, Belkhode et al.’s protocol sets the stage for an insightful comparison between two promising OSA treatments. This study has the potential to propose a new therapeutic option for moderate OSA, though the noted data discrepancy should be addressed to maintain the scientific rigor of the study’s findings. Additionally, exploring patient tolerance and adherence in more depth could yield further insights, especially in evaluating the long-term potential of CMOA as a comfortable, effective alternative to MADs in OSA management. Understanding the underlying mechanism of action for maxillary appliances and addressing the issue of adjustability will be essential in assessing the true clinical viability of CMOA as an innovative therapeutic option.

## Data Availability

Not applicable.

## Abbreviations

AHI: Apnea/hypopnea index
CMOA: Customized maxillary oral appliance
CPAP: Continuous positive airway pressure
MAD: Mandibular advancement device
OSA: Obstructive sleep apnea
TMJ: Temporomandibular joint
